# Developing and evaluating an evidence-based practice research competency enhancement program for clinical nurses in Korea: a pilot study

**DOI:** 10.1186/s12912-024-01749-8

**Published:** 2024-03-03

**Authors:** Suhyun Kim, Hye Won Jeong

**Affiliations:** 1 Department of Nursing, Mokpo National University, 1666 Yeongsan-ro, Cheonggye-myeon, Muan-gun, Jeollanam-do 58645 Republic of Korea; 2 Department of Nursing, Nursing Education Team, Chonnam National University Hospital and College of Nursing, Chonnam National University, 42 Jebong-Ro, Dong-Gu, Gwangju, 61469 Republic of Korea

**Keywords:** Evidence-based practice, Mentors, Pilot projects, Mixed method

## Abstract

**Background:**

Evidence-based practice (EBP) is crucial for delivering high-quality healthcare and effective self-care. Enhancing clinical nurses’ research competencies through structured mentorship is key to the widespread application of EBP. This study evaluated a newly developed Research Competency Enhancement Program (RCEP), aimed at bolstering EBP among experienced nurses.

**Methods:**

Conducted in a tertiary university hospital in Korea, this single-group study employed a pretest-post-test design and a mixed-methods approach. The RCEP involved 11 experienced clinical nurses in an 8-week intervention, featuring mentor-led workshops, interactive sessions, and resource-driven activities. Data were collected using the Evidence-Based Practice Beliefs Scale (EBPB), the Evidence-Based Practice Attitude Scale (EBPA), and the Research Practice Ability (RPA) tool, alongside qualitative feedback. These measures assessed the program’s feasibility, acceptability, and preliminary effectiveness.

**Results:**

The quantitative analysis indicated significant improvements in research competency post-intervention. Mean scores on the EBPB and RPA scales increased (Z = -2.53, *p* = .011; Z = -2.66, *p* = .008). Participants described the RCEP as inspirational and challenging, creating an environment conducive to research. Facilitators included mentor support and innovative learning tools, while barriers were internet connectivity and scheduling conflicts. Suggestions for improvement included more hands-on sessions, small team collaborations, and integration with academic institutions.

**Conclusion:**

The RCEP, facilitated by EBP mentors, significantly improved the research competencies and attitudes of clinical nurses towards EBP. The study underscores the importance of continual RCEP refinement, integrating structured, interactive, and collaborative elements to further empower nurses in evidence-based practice. The program shows promise in enhancing research competencies and fostering a commitment to EBP in clinical settings.

**Supplementary Information:**

The online version contains supplementary material available at 10.1186/s12912-024-01749-8.

## Background

Nursing research plays a pivotal role in enhancing healthcare service quality [1]. The involvement of nurses in research activities not only enriches their professional expertise but also significantly contributes to the development of a robust nursing knowledge base. This advancement positively impacts patient safety, reduces medical costs, and improves overall care quality [1, 2]. However, the complex and demanding nature of clinical settings often impedes the practical application of research findings in nursing care. It is in this context that the gap between theoretical knowledge and practical application becomes apparent, underscoring the importance of evidence-based practice (EBP).

EBP combines the most reliable research evidence with nurses' clinical skills, patient preferences and values, and the resources at hand to guide nursing decisions [2]. Acquiring skills in EBP involves systematic literature reviews and extensive training. This training is necessary to formulate clinical questions, search through both domestic and international databases, critically evaluate the literature, and make informed recommendations [3, 4]. Despite these requirements, clinical nurses frequently encounter challenges in applying EBP. These challenges stem from a lack of knowledge and skills to effectively use evidence-based information, organizational cultures that do not support EBP, and the absence of mentorship [5, 6]. Consequently, many nurses rely on outdated information from old policies, procedures, and traditional educational programs, rather than utilizing current research-based evidence in their clinical practice [7].

To effectively bridge this gap, the role of EBP mentors and the significance of Research Competency Enhancement Programs (RCEP) must be emphasized. When integrated properly into hospital systems, EBP mentors provide crucial support and learning opportunities, assisting nurses in developing and honing their EBP skills [5]. Additionally, organizational structures should be aligned to support these mentorships and ensure continuous EBP training resources [8]. The Advancing Research and Clinical Practice through Close Collaboration (ARCC) model demonstrates that nursing EBP beliefs and practices improve markedly when organizational culture endorses EBP, identifies barriers and facilitators, and employs mentors efficiently [2].

Despite recognizing the importance of EBP and mentoring in nursing, there is a significant scarcity of studies aimed at enhancing the EBP research performance of clinical nurses in Korea. Most research focuses on factors related to EBP rather than the impact of comprehensive programs or the influence of mentors in facilitating EBP [9, 10]. Experimental studies have explored the effects of mentor education programs and formal skill-building initiatives, but there is still a need for a structured, theory-based approach to enhancing EBP skills [8, 11]. This approach aims to provide essential data for the proliferation and advancement of EBP in nursing practice, particularly underscoring the vital roles of EBP mentors and structured, programmatic methods for developing EBP skills [2].

The ARCC model is pivotal in fostering, executing, and maintaining EBP at the organizational echelon [2]. This model involves assessing the organization’s readiness and cultural alignment for EBP implementation, pinpointing strengths, challenges, and EBP mentors, applying evidence within the organization, and appraising outcomes based on tangible changes. In the realm of evidence-based practice, tools for organizational-level measurement have proven beneficial within academic contexts [12], seamlessly bridging nursing education and practical application. In our study, organizational traits were discerned through comprehensive interviews. The role of EBP mentors in supporting a clinician’s EBP adoption is crucial, as it cultivates an organizational ethos conducive to EBP and bolsters an individual’s confidence in evidence-based methodologies. Our study incorporates a theoretical framework that focuses on beliefs and attitudes towards EBP as individual-level characteristics, integrating them within the ARCC model's principles. The research questions we seek to answer are:

- What impact does the RCEP have on clinical nurses’ competencies in EBP?
- How do nurses describe their experiences while participating in the RCEP?

## Methods

### Study design

**Fig. 1:**
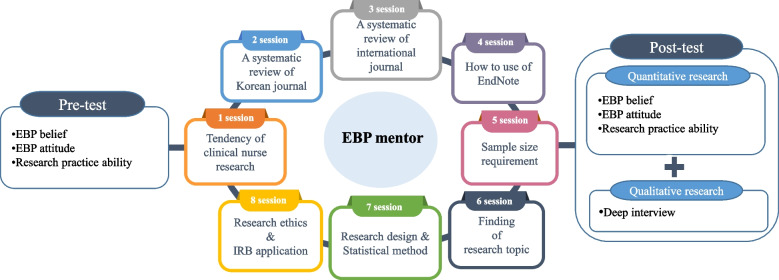
RCEP based on the ARCC model

This study utilized a mixed-methods approach, specifically employing Creswell and Clark’s explanatory design [13], to develop and verify the effectiveness of an RCEP for clinical nurses. The study was executed in two phases: the initial phase involved the collection of quantitative data through a single-group pre-and-post experimental design. This phase aimed to evaluate changes in the participants’ EBP beliefs, attitudes, and research practice skills. The subsequent phase entailed gathering qualitative data via content analysis of participant interviews. This phase aimed to investigate their experiences and perceptions of the RCEP, thus providing context and deeper insight into the quantitative results (Fig. 1).

### Participants and setting

The study involved 11 nurses from a tertiary university hospital in Korea. The inclusion criteria required nurses to understand the study’s purpose and consent to participate, hold a master’s degree (excluding those who wrote a master’s thesis), and possess over three years of clinical experience. The sole exclusion criterion was refusal to participate, with the option for participants to withdraw at any time. A clinical nurse educator, holding a doctoral degree in nursing, provided mentorship throughout the study.

### Sample size

The sample size was determined using a nonparametric test with G*Power 3.1.9.4. This calculation was based on the effect size reported by Yoo and Kim [14], a significance level of α = 0.05, an effect size of *d* = 0.97, and a power of 1—ß = 0.80, resulting in a total of 11 participants. Anticipating a 20% dropout rate, 13 nurses were initially recruited. However, two withdrew, leaving data from 11 participants for quantitative analysis. Following the program, nine participants were interviewed to collect and analyze qualitative data.

### Intervention

#### RCEP development

The RCEP, designed for clinical nurses and based on the ARCC model, positioned the EBP mentor as central and included eight sessions. A preliminary educational needs survey revealed that the most cited reason for delayed thesis writing among participants was “difficulty selecting a research topic” (63.6%), with “difficulty reviewing the literature” (18.2%) as the second most common. To address these issues, the program offered content aimed at assisting participants in identifying research topics via literature reviews. The program consisted of hourlong sessions focusing on the following topics: session 1, clinical nursing research trends: overview of current and emerging trends; session 2, systematic literature reviews of domestic journals: techniques and strategies for effective review; session 3, systematic literature reviews of international journals: expanding search and analysis globally; session 4, EndNote 20: training on utilizing this reference management software; session 5, G*Power analysis: guidance on using this tool to determine sample sizes; session 6, the identification of nursing research topics in the clinical field: strategies to pinpoint relevant and impactful research areas; session 7, research design and statistical methods: overview of various research designs and statistical methods pertinent to nursing research; and session 8, research ethics and Institutional Review Board application methods: critical legal and ethical considerations and procedural guidance for applications. The sessions’ format varied, accommodating different learning styles: session 1 was a lecture, sessions 2 through 5 included a mix of lectures and practical exercises, session 6 involved a discussion, and sessions 7 and 8 were lecture-based. The program’s structure was validated for content by a nursing professor, a nursing education team leader with a master’s degree in nursing, and three clinical nurse educators holding master’s degrees. The content validity index (CVI) achieved was 0.99.

#### RCEP application

This study was conducted over eight sessions at biweekly intervals from September 2021 to January 2022. The implementation consisted of interactive, practical sessions conducted in the education room of the research hospital. Participants, who were nurses, were asked to bring their personal laptops to support hands-on learning. Each session was followed by a two-week period dedicated to self-study and review. This interval allowed participants to integrate their new knowledge and apply their skills in practical settings. Throughout this period, participants were encouraged to engage in ongoing communication with the EBP mentor for guidance and clarification. This mentorship was conducted via an online platform, where participants could post queries or reflections as research notes after each session. These notes had a twofold purpose: they reinforced learning and provided data for the evaluation and enhancement of the program.

### Measurements

Data from 11 participants were analyzed for the pretest and post-test surveys. Two participants who withdrew from the study were excluded. The study investigated general characteristics of the subjects, including age, gender, marital status, work experience, and department.

#### EBP beliefs

The Korean version of the Evidence-Based Practice Beliefs Scale (EBPB), developed by Melnyk et al. [15], was employed to assess EBP beliefs in our study. This 16-question instrument evaluated support for EBP value and confidence in implementing evidence-based practice, using a 5-point Likert scale that ranged from 1 (very negative) to 5 (very positive). A higher score indicated a stronger belief in EBP. Regarding the tool’s reliability, Cronbach’s α was reported as 0.85 in Melnyk et al.’s study [15] and 0.84 in the current study.

#### EBP attitudes

The Korean version of the Evidence-Based Practice Attitude Scale (EBPA), developed by Aarons [16], was utilized to assess attitudes towards EBP. This instrument consists of 15 items across four subareas: requirement, openness, appeal, and divergence, with three questions in requirement and four in each of the latter three areas. Responses were measured on a 5-point Likert scale, ranging from 0 (not at all) to 4 (very much). Higher scores indicated more positive attitudes towards EBP. Cronbach’s α was reported as 0.96 in Aarons' study [16] and 0.81 in the current study.

#### Research practice ability

Research practice ability (RPA) was evaluated by adapting and augmenting the 12 domains of the Program Outcome Self-Assessment Tool in Korean Nursing Baccalaureate Education, as developed by Kim [17]. This tool encompasses seven items, each rated on a 4-point Likert scale from 1 (very difficult) to 4 (very easy). Higher scores signified greater research competency. In Kim’s study [17], the tool’s Cronbach’s α was 0.86, compared to 0.92 in this study.

### Data collection

#### Quantitative data collection

The study involved clinical nurses who had completed a master’s degree program without a thesis over two weeks in August 2021. Participants expressed their willingness to participate and provided informed consent. Data collection spanned from September 2021 to January 2022. For quantitative data, a pre-survey was administered to the intervention group just before the program started, and a post-survey was conducted immediately after its conclusion. A research assistant collected the completed surveys.

#### Qualitative data collection

In January 2022, qualitative data were collected using in-depth, unstructured interviews, following the conclusion of the program. Participants were given the freedom to choose the time and location for their interviews. The interviews were conducted by a researcher, who is also a professor of nursing and an expert in qualitative research methods, on a voluntary basis. All participants elected to conduct their interviews via non-face-to-face means, utilizing Zoom (Zoom Video Communications Inc., San Jose, CA, USA). With prior consent, these interviews were recorded and transcribed by the researcher within a 24-h period. The primary question posed during these interviews was: “What was your experience participating in the RCEP?” This was supplemented by additional inquiries such as, “What helped you to participate in the RCEP,” “What were the barriers to your participation in the RCEP,” and “What are your overall evaluations and suggestions for RCEP?” Each interview session lasted between 60 to 80 min (Supplement).

### Data analysis

Quantitative data were analyzed using SPSS/Windows 25.0 (SPSS Inc., Chicago, IL, USA). A statistical significance threshold was set at α = 0.05. The reliability of the measuring tool was assessed using Cronbach’s α coefficient. We utilized descriptive statistics to examine participants’ general characteristics, EBP beliefs and attitudes, and research competency. Due to the limited sample size, the normality assumption was not met. Consequently, the Wilcoxon signed rank test was employed to assess the differences in scores of the dependent variable. To analyze the emotions and learning points recorded by nurses following each educational session, keyword frequency analysis was conducted, and WordCloud (Zygomatic, Vianen, The Netherlands) was used for visual representation.

For the qualitative data, we applied the content analysis method of Hsieh and Shannon [18]. The transcribed interview data were managed using NVivo 12 (QSR International, Burlington, MA, USA). Semantic units of the data guided classification and coding processes. Two researchers independently reviewed the transcribed data to comprehend the implications of participating in the RCEP. We extracted meaningful words or sentences, integrating and coding similar content into subcategories. These subcategories were then grouped into higher categories based on common content relationships. Final results were established after collaborative discussions between the researchers.

### Rigor

To ensure a reliable and valid qualitative study, the stringent evaluation criteria outlined by Lincoln and Guba [19] were adhered to. The truth value was maintained by continually comparing interview content with transcription data, ensuring consistency between them. Applicability was assessed by presenting the study’s findings to two nurses who were program participants but not interviewees, to verify if the results resonated with their experiences. For consistency, the data analysis process and procedures were rigorously followed. The research process and outcomes were also reviewed by three qualitative research experts to validate these aspects. Neutrality was upheld by minimizing preconceptions and biases as much as possible. Post-interview, analytical memos were utilized for reflection and introspection.

### Ethical considerations

The research was conducted with the approval of the Institutional Review Board of Chonnam National University Hospital (CNUH-2021–425). Participants received detailed information about the research objectives, data collection procedures, and the confidentiality of their personal information. They were also provided with consent forms.

## Results

### General characteristics of the participants

The study participants’ general characteristics are presented in Table 1. Among them, eight (72.7%) were aged 40 or older, seven (63.6%) worked on the ward, and seven (63.6%) had completed their master’s degree program in under five years.

**Table 1:** Participants’ general characteristics

Characteristic	Category	(*n* = 11)	
		***n*** (%)	**Mean (SD)**
Age (years)	≤ 40	3 (27.3)	44.63 (6.23)
	> 40	8 (72.7)	
Gender	Men	0 (0)	
	Women	11 (100.0)	
Total career length (years)	≤ 20	3 (27.3)	21.55 (6.35)
	21–25	5 (45.4)	
	≥ 26	3 (27.3)	
Work unit	Ward	7 (63.6)	
	Other (OR, PACU, IRD)	4 (36.4)	
Duration of postgraduate degree (years)	≤ 5	7 (63.6)	5.18 (2.64)
	> 5	4 (36.4)	

*SD* standard deviation, *OR* operating room, *PACU* post-anesthesia care unit, *IRD* insurance review department

### The ARCC-based RCEP effect

The RCEP’s impact on participants was assessed by evaluating changes in EBPB, EBPA, and RPA, as shown in Table 2. A significant increase in the EBPB score was observed, rising from 3.48 (± 0.41) pre-intervention to 4.03 (± 0.49) post-intervention, reflecting enhanced belief in EBP’s value (Z=-2.53, *p* = .011). RPA scores also exhibited notable improvement, moving from 3.31 (± 0.59) to 4.12 (± 0.74), indicating heightened research proficiency (Z=-2.66, *p* = .008). However, while there was an increase in the EBPA score post-intervention, this change was not statistically significant, pointing to potential areas for further refinement in the program.

**Table 2:** Effects of RCEP on variables

Variable	(*n* = 11)			
	**Pretest**	**Post-test**	**Z**	***p***
	**Mean (SD)**	**Mean (SD)**		
Evidence-Based Practice Beliefs Scale (EBPB)^a^	3.48 (0.41)	4.03 (0.49)	-2.53	.011
Evidence-Based Practice Attitudes Scale (EBPA)^a^	2.81 (0.37)	3.02 (0.67)	-0.48	.635
Openness^a^	2.80 (0.57)	3.14 (0.77)	-0.82	.410
Appeal^b^	2.73 (0.45)	2.52 (1.09)	-0.63	.531
Divergence^a^	2.82 (0.42)	3.18 (0.58)	-1.69	.091
Requirements^a^	2.94 (0.47)	3.33 (0.52)	-1.26	.208
Research Practice Ability (RPA)^a^	3.31 (0.59)	4.12 (0.74)	-2.66	.008

*SD* standard deviation

^a^indicates that "the ranking is based on negative ranking"

^b^indicates that "the ranking is based on positive ranking"

### Feelings and lessons after RCEP: keyword frequency analysis

**Fig. 2:**
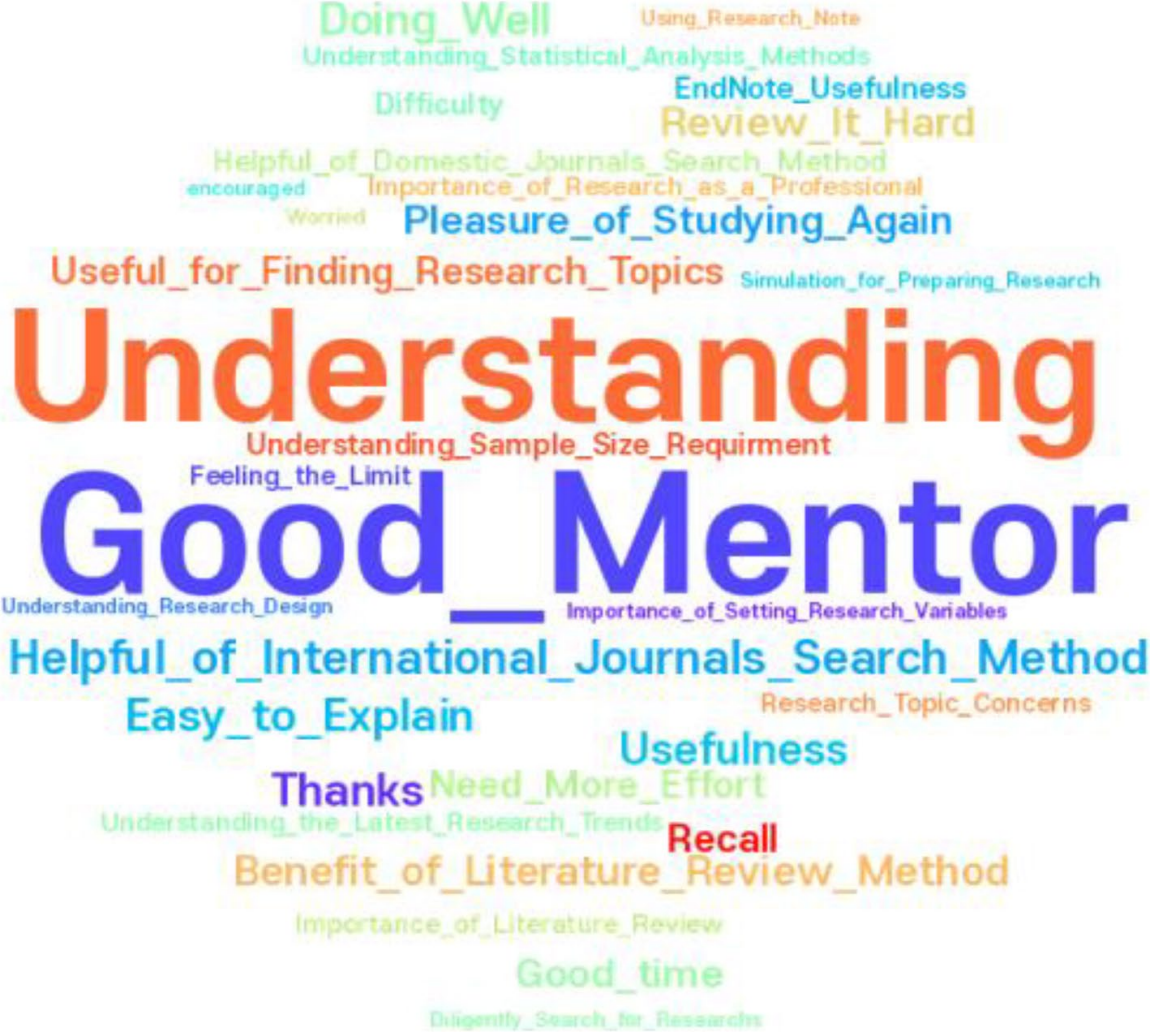
Feelings and learnings following RCEP through frequency analysis of keywords

A keyword frequency analysis, depicted in Fig. 2, shed light on the participants’ emotional and educational experiences following the RCEP. From 102 responses, 159 keywords were identified. Dominant themes emerged around the importance of a “good mentor” and the effectiveness of techniques for “international journal searches,” highlighting recognition of mentorship and practical skill acquisition. Participant feedback included expressions of gratitude toward the teacher, who served as both mentor and lecturer, for detailed explanations. Comments included: “I was grateful for the teacher, who is both a mentor and lecturer, for explaining information in detail.” “I am very grateful for the opportunity to listen to such a good lecture.” “I expect that my understanding of research will increase.” “The method of searching international journals was really useful.” “I understood the lecturer’s straightforward and detailed explanations.” These direct quotations from participants underscore a sense of gratitude, enhanced comprehension, and the practical application of the learned content.

### Content analysis of participants’ experience with RCEP

The analysis of interviews concerning participants’ experiences with RCEP yielded four main categories and 16 subcategories: “meaning of the educational experience,” “factors facilitating RCEP participation,” “barriers to participation,” and “modifications for future RCEP operation” (Table 3).

**Table 3:** Content analysis of participants’ RCEP experience

Category	Subcategory	Quotation
Meaning of the educational experience	Lighthouse of my heart	“*This education has been a guide for how I should conduct my research. It has been more than 10 years since I had completed my master’s degree program, and I have been wondering where to start for a long time*.”
	Inner stimulant	“*A nurse like me became a mentor and led the entire schedule, which really motivated me. Participating in the educational program gave me confidence that I could conduct research too*.”
	Power to challenge myself	“*While working as a clinical nurse for 25 years, I fully felt that research was important to nursing, but I could not do it alone. This training gave me the strength to take on challenges*.”
Factors facilitating RCEP participation	Full support and encouragement of mentors	“*It was because of the interest and support of the mentor that I was able to participate in the educational program until the end. When the training day approached, they notified us of the schedule through text message and group KakaoTalk, and they checked our progress*.”
	Interest from colleagues	“*Since we participated in the training together, we discussed each other’s areas of interest and shared our progress, so I thought I should work harder too. In particular, I received more help when collaborating as a team of four or five people*.”
	Use of new learning tools	“*As your education progresses, there are steps to take according to your research topic. When I post my areas of interest and questions on the Padlet’s large window, the content is created like a Post-It, and comments from mentors and colleagues are added. Although it is my research topic, it was a great source of strength to think about it together rather than worrying alone*.” “*In fact, we are a culture where no one will ask a question during a lecture. I liked that posting questions on the Padlet was much less burdensome, and the mentor personally answered them*.”
	Clinical nurse educators serving as assistants	“*Depending on the lecture topic, more nurse educators were added to help. Those like me who were unable to follow the lecture were assisted separately. I would have given up if I had been alone*.”
Barriers to participation	Unstable internet connections at workplaces	“*It was time to learn how to download and use the EndNote program. When I connected to the same place at the same time and downloaded it, it didn’t work properly*.”
	Limited literature search databases	“*It has been a while since I completed my master’s degree program, so it’s hard for us to access the literature search database for a fee. All the participants shared and used one mentor ID*.”
	Problems with individual laptop specifications	“*I practiced while following the mentor’s lecture, but my laptop was old, so I missed the lecture because it took a lot of buffering time.*”
	Education schedules overlapping with hospital events or external schedules	“*It is officially education time, but during this period, the hospital’s electronic medical record system was completely reorganized, and my colleagues were struggling with their work. There were days when I could not attend the program*.” “*The unit manager told me to go to the educational session, but I could not because it was the day of an infection control event at the hospital*.”
Modifications for future RCEP operation	Appropriate sessions and times	“*I am very satisfied with the training taking place every two weeks. Two weeks was enough time for me to think about what I had learned with little psychological burden and to find my research topic*.” “*I really liked the opportunity to learn about research for about an hour after work. If the lecture time had been long, I think I would have been tired*.”
	Impressive new research methods	“*When I was studying research methods in my master’s course, I never heard of network analysis. It was impressive to learn about a new research methodology in this program*.”
	Preference for face-to-face lectures	“*Due to the COVID-19 situation, many educational sessions aren’t taking place face-to-face. I hope that older people like us will continue to give face-to-face lectures as they do now. We could not concentrate when we used Zoom*.”
	Preference for small teams	“*I think that if every four to five nurses with a will for research were assigned a professional mentor who continued to manage them, they would have achieved good results after completing the program.*”
	Request for collaborative research opportunities with professors at nursing colleges and hospitals	“*In fact, nursing college professors are the ones who need to do research. While we are in practice, we have many research topics, but it is difficult to develop them into research. I hope there will be a forum where professors and nurses can regularly discuss their fields of interest*.”

#### Meaning of the educational experience

This category comprised subcategories like “lighthouse of my heart,” “inner stimulant,” and “power to challenge again.” Many participants had not completed their master’s degree courses for a considerable period, turning research into a daunting task. RCEP instructed participants in specific methods for initiating research in the clinical field. Mentors played a crucial role in building confidence, acting as a catalyst for participants to commence their research projects. RCEP underscored the program’s transformative and motivational aspects, highlighting the participants’ renewed confidence and direction in conducting clinical research.

#### Factors facilitating RCEP participation

This category included four subcategories: “full support and encouragement of mentors,” “interest from colleagues,” “use of new learning tools,” and “clinical nurse educators as assistants.” Even after the commencement of RCEP, participants expressed concerns about their ability to continue due to work commitments. Mentors consistently communicated RCEP schedules and progress via text messages and social network services. Engaging in discussions about shared interests and progress with other RCEP participants played a significant role in maintaining their engagement throughout the program. Initially, the “Padlet” program, introduced by an RCEP mentor for individual research topic consultations, appeared challenging, but its real-time feedback feature through comments proved beneficial. Besides mentors, the clinical nurse educators who supported the participants’ learning were instrumental in ensuring smooth progress and active participation. The combination of mentor support, peer interaction, innovative learning tools, and assistance from clinical nurse educators created a conducive and stimulating learning environment.

#### Barriers to participation

This category included four subcategories: “unstable internet connections at workplaces,” “limited literature search databases,” “problems with individual laptop specifications,” and “education schedules overlapping with hospital events or external schedules.” Unstable internet connections were prevalent when multiple users occupied a single space, as experienced in the RCEP. Moreover, participants were unable to utilize the hospital’s subscription for a specialized literature search database. Consequently, they relied on sharing the mentor’s university subscription service credentials. The absence of a designated area in the hospital for conducting literature searches using PCs further complicated the situation. Participants often used their personal laptops, leading to compatibility issues during the RCEP sessions. Additionally, scheduling conflicts arose as the RCEP coincided with hospital events or nursing department management meetings, preventing some participants from attending. These barriers highlight the significant logistical and resource-related challenges faced during the program.

#### Modifications for future RCEP operation

This category included the subcategories of “appropriate sessions and times,” “impressive new research methods,” “preference for face-to-face lectures,” “preference for small teams,” and “request for collaborative research opportunities with professors at nursing colleges and hospitals.” Participants expressed high satisfaction with the current eight-day lecture schedule, which spanned approximately one hour per session. They particularly appreciated the program’s ability to facilitate efficient use of their post-work hours without inducing boredom. A notable interest was observed in the network analysis research method, introduced for the first time in the RCEP. Regarding the format, participants showed a clear preference for in-person lectures, which allowed direct support from clinical nurse educators, over online lectures known for their repetitive nature. Additionally, the nurses reported high satisfaction with the small-group learning approach adopted by the intervention. Their engagement in the RCEP highlighted the variety of research topics relevant to their clinical environments and sparked an interest in pursuing collaborative research with hospitals and nursing college professors. Improvement suggestions varied, encompassing logistical adjustments, format preferences, and collaborative opportunities, all indicating a collective aspiration for more efficient, engaging, and contextually relevant learning experiences.

## Discussion

This study’s mixed-methods approach facilitated a comprehensive and integrated analysis of the ARCC model-based program's influence on improving clinical nurses’ research competencies. The quantitative data demonstrated notable advancements in EBP beliefs and research competencies among the participants, highlighting the program’s success in strengthening both the practical and psychological dimensions of research-oriented practice. Meanwhile, the qualitative data offered more profound insights into these developments, encapsulating the participants’ experiences, challenges faced, and the pivotal role of mentorship in enhancing their journey towards improved research competency.

The participants in this study were clinical nurses who had completed a master’s degree program and subsequently engaged in an intervention to compose a master’s thesis. These nurses initially lacked the information literacy skills required for tasks such as conducting a literature review and selecting a research topic. In each educational session, their skills were individually assessed and personalized feedback was provided. The quantitative phase of the study revealed significant enhancements in the participants’ EBP beliefs and research competencies, which were in line with the study’s objectives. This progress highlights the effectiveness of structured, mentor-led programs in equipping nurses with the necessary skills and confidence for research involvement. A notable increase in EBP beliefs and competencies was observed [11], corroborating the findings of this study. Unlike the team-based research program examined by Gorsuch et al. [11], our program focused on enhancing the research skills of individual clinical nurses. For the successful integration of EBP in clinical settings, it is essential to establish an organizational culture with a focus on EBP mentors and to provide support enabling clinical nurses to actively engage in EBP [11]. As EBP mentors, clinical nurse educators can bolster nurses’ confidence in EBP usage and instruct them on its practical application [20]. Among the nurses in this study, 36.4% completed a master’s thesis and demonstrated improved research competencies. The ARCC model suggests that organizational readiness and culture can act as either facilitators or obstacles in the implementation of EBP [21]. This study lays the groundwork for fostering an EBP culture within nursing organizations and for the development of the participants’ individual skills.

Participants’ attitudes toward EBP showed an increase compared to their pre-program levels, although the change was not statistically significant. This finding aligns with a study on online and face-to-face EBP courses for nurses, where improvements in EBP skills and knowledge were noted, but attitudes and practices remained unchanged [22]. The difficulty in altering EBP attitudes through education stems from various influences, including geographic and cultural factors, professional environments, and organizational cultures [22]. Notably, Korean hospital organizations exhibit comparatively lower EBP attitudes than those in other countries [23]. Furthermore, the study participants, averaging 21.6 years of clinical experience, relied heavily on their personal expertise and knowledge, displaying a pronounced preference for existing practices [24]. Future research should focus on nurses at different stages of their clinical careers.

Qualitatively, participants reported increased confidence and motivation for research, largely attributing this growth to the mentorship and guidance received. These observations are crucial for grasping the program’s personal impact and the subtle shifts in mindset it induces. The in-depth interviews highlighted both the empowering aspects of the RCEP and the systemic and individual obstacles encountered, consistent with wider EBP challenges identified in the literature [2, 5]. The RCEP aims to enhance patient care by improving clinical nurses’ research topic identification, master’s degree preparation, and integration of EBP into practice. Given that EBP mentor programs offer significant cultural and financial benefits to medical institutions [21], a concerted institutional effort is required to gradually overcome barriers and foster an EBP culture in the long term. Additionally, career nurses unfamiliar with literature searches found EBP classes more approachable with the support of clinical nurse educators acting as assistants. An EBP mentor team is crucial in cultivating and implementing a supportive EBP culture within organizations. Moreover, as indicated in the study’s in-depth interviews, incorporating nursing professors into the EBP mentor team is essential for industry-academic collaboration between hospitals and nursing colleges.

The findings of this study have significant implications for both nursing education and practice. In the educational context, these findings support the incorporation of structured mentorship and hands-on skill training into nursing curricula, with a focus on developing both EBP competencies and attitudes. From a practical standpoint, the study highlights the importance of fostering supportive organizational cultures and providing sufficient resources to encourage nurses’ involvement in research. Establishing a culture of EBP necessitates dedication from nursing leadership and institutional backing, which includes investment in training, mentorship, and resource distribution.

This study is limited by its single-group, before-and-after design, lacking a control group, which restricts the ability to definitively ascertain the program’s effectiveness. The relatively small sample size of 11 nurses diminishes the statistical power and limits the generalizability of the findings, necessitating caution in interpreting the results. The study’s focus on experienced clinical nurses from a single university hospital may not accurately represent the broader nurse population or different settings, further limiting generalizability. Additionally, restricted access to a wide range of literature databases may have affected the depth of theoretical understanding and discussion. Issues with internet connectivity might have impacted the participants’ engagement and the overall success of the research competency enhancement program, underscoring the need for reliable technological infrastructure in online educational and research initiatives. These limitations should be carefully considered when interpreting the study’s findings and in the design of future research.

## Conclusion

In this study, by applying the ARCC model and a mixed-methods approach, we developed and evaluated a program aimed at enhancing the research competency of individual clinical nurses, differing from conventional team-based approaches. The results suggest improvements in nurses’ EBP beliefs and research competencies, highlighting the importance of leadership support and resource allocation for fostering an EBP culture. Although the findings are encouraging, the impact of the RCEP program needs further exploration due to the study’s limitations. Future research should involve diverse and larger samples of nurses and include control groups to reinforce these findings and offer a clearer understanding of the program’s effectiveness in enhancing nursing research competencies.

### Supplementary Information

**Additional file 1:** **Supplement.** Guideline for interview questions regarding the experiences of nurses participating in the RCEP used during the interview sessions with the participants.

## Data Availability

The datasets used and/or analyzed during the current study are available from the corresponding author (y2k331646@gmail.com) upon reasonable request.

## Abbreviations

EBP: Evidence-based practice
RCEP: Research competency enhancement program
EBPB: Evidence-based practice belief
EBPA: Evidence-based practice attitude
RPA: Research practice ability
ARCC: Advancing research and clinical practice through close collaboration
