# Supplementary material for: Developing and evaluating an evidence-based practice research competency enhancement program for clinical nurses in Korea: a pilot study
Source: BMC Nurs. 2024 Mar 3;23:111. doi: 10.1186/s12912-024-01749-8 (PMC10909249; doi:10.1186/s12912-024-01749-8)
Supplement: Supplementary file 1 — Additional file 1: Supplement. Guideline for interview questions regarding the experiences of nurses participating in the RCEP used during the interview sessions with the participants. [file 12912_2024_1749_MOESM1_ESM.docx]

Guideline of Questions for Interview

**Supplement.** Guideline for interview questions regarding the experiences of nurses participating in the RCEP used during the interview sessions with the participants.

| **Category** | **Questions** |
| --- | --- |
| Introduction | Please feel free to discuss your experiences participating in the RCEP.  What motivated you to participate in the RCEP? |
| Development | Could you please share your experiences participating in the RCEP?  What aspects did you enjoy about your participation in the RCEP?  What does your participation experience in the RCEP mean to you?  What are the significant implications of your participation experience in the RCEP?  What challenges did you encounter, and how did they provide assistance?  Is there anything else you would like to add regarding the RCEP?  How would you evaluate the RCEP? |
| Conclusion | Would you recommend other nurses to participate in the RCEP in the future?  Please feel free to share any additional comments you may have about the RCEP. |
